# Urogenital schistosomiasis in Cabo Delgado, northern Mozambique: baseline findings from the SCORE study

**DOI:** 10.1186/s13071-017-2592-8

**Published:** 2018-01-10

**Authors:** Anna E. Phillips, Pedro H. Gazzinelli-Guimarães, Herminio O. Aurelio, Neerav Dhanani, Josefo Ferro, Rassul Nala, Arminder Deol, Alan Fenwick

**Affiliations:** 1 Schistosomiasis Control Initiative, Department of Infectious Disease Epidemiology, Imperial College London, W2 1PG, London, UK; 2 Faculdade of Health Sciences, Universidade Católica de Moçambique (UCM), Beira, Mozambique; 3 Ministerio da Saúde, Maputo, Mozambique

**Keywords:** Schistosomiasis, *Schistosoma haematobium*, School-based treatment, Community-wide treatment, Mozambique

## Abstract

**Background:**

The results presented here are part of a five-year cluster-randomised intervention trial that was implemented to understand how best to gain and sustain control of schistosomiasis through different preventive chemotherapy strategies. This paper presents baseline data that were collected in ten districts of Cabo Delgado province, northern Mozambique, before treatment.

**Methods:**

A cross-sectional study of 19,039 individuals was sampled from 144 villages from May to September 2011. In each village prevalence and intensity of *S. haematobium* were investigated in 100 children first-year students (aged 5–8 years), 100 school children aged 9–12 years (from classes 2 to 7) and 50 adults (20–55 years). Prevalence and intensity of *S. haematobium* infection were evaluated microscopically by two filtrations, each of 10 ml, from a single urine specimen. Given that individual and community perceptions of schistosomiasis influence control efforts, community knowledge and environmental risk factors were collected using a face-to-face interview. Data were entered onto mobile phones using EpiCollect. Data summary was made using descriptive statistics. Chi-square and logistic regression were used to determine the association between dependent and independent variables.

**Results:**

The overall prevalence of urogenital schistosomiasis was 60.4% with an arithmetic mean intensity of infection of 55.8 eggs/10 ml of urine. Heavy infections were detected in 17.7%, of which 235 individuals (6.97%) had an egg count of 1000 eggs/10 ml or more. There was a significantly higher likelihood of males being infected than females across all ages (62% *vs* 58%; *P* < 0.0005). Adolescents aged 9–12 years had a higher prevalence (66.6%) and mean infection intensity (71.9 eggs/10 ml) than first-year students (63.1%; 58.2 eggs/10 ml). This is the first study in Mozambique looking at infection rates among adults. Although children had higher levels of infection, it was found here that adults had a high average prevalence and intensity of infection (44.5%; 23.9 eggs/10 ml). Awareness of schistosomiasis was relatively high (68.6%); however, correct knowledge of how schistosomiasis is acquired was low (23.2%) among those who had heard of the disease. Schistosomiasis risk behaviour such as washing (91.3%) and bathing (86.7%) in open water sources likely to be infested with host snails was high.

**Conclusions:**

Urogenital schistosomiasis is widespread in Cabo Delgado. In addition, poor community knowledge about the causes of schistosomiasis and how to prevent it increases the significant public health challenge for the national control program. This was the first study in Mozambique that examined infection levels among adults, where results showed that *S. haematobium* infection was also extremely high. Given that this controlled trial aims to understand the impact of different combinations of schistosomiasis control through treatment of communities, schools, and treatment holidays over a five-year period, these findings highlight the importance of examining the impact of different treatment approaches also in adults.

**Trial registration:**

The trials have been registered with the International Standard Randomised Controlled Trial registry under ISRCT 14117624 Mozambique (14 December 2015).

## Background

Schistosomiasis affects at least 240 million people worldwide, with an estimated 20 million suffering from severe and debilitating forms of the disease [1, 2]. The burden is concentrated in Africa, where more than 90% of the infections occur [3]. Schistosomiasis is strongly associated with poverty, where the disease delays the social and economic development of endemic countries [4, 5].

Schistosomiasis is a major public health problem in Mozambique, as shown by an epidemiological survey of schistosomiasis and soil-transmitted helminths (STH) among school children carried out between 2005 and 2007 [6]. The mean estimated prevalence of urogenital schistosomiasis, *Schistosoma haematobium*, was 47% but schistosomiasis is focal, and so prevalence varied dramatically across the country [6]. In Cabo Delgado Province, the chosen area for this study, the prevalence was 57.9% ranging from 8.8% on the coast to 93% inland [6]. In contrast, the prevalence of intestinal schistosomiasis, whose causative agent is *S. mansoni*, is extremely low in Mozambique, with an average prevalence of 1% [6–8].

Regular large-scale preventive chemotherapy with praziquantel is the current control strategy recommended by the World Health Organisation (WHO) that aims to alleviate subtle morbidity and prevent infected individuals from developing severe, late-stage morbidity due to schistosomiasis [9]. The current recommended WHO treatment strategy for schistosomiasis depends on the prevalence among school-aged children (SAC) aged 5–14 years, where if infection is higher than 50% the school-aged population should be treated once a year; if the prevalence is between 10 and 50% treatment is focused on SAC biennially; and if less than 10% SAC should be treated twice, once at school entry and once before finishing school [10]. Studies have found, however, a community-wide approach for schistosomiasis a very effective strategy for reaching children who do not attend school as well as potentially high-risk adults [11–13]. Recent models on STH have also demonstrated the potential cost and health benefits for expansion of drug coverage to the entire community [14, 15].

The Schistosomiasis Consortium for Operational Research and Evaluation (SCORE) was established in 2008 to provide an evidence base for programme managers to address strategic questions about schistosomiasis control. It comprises of multi-country field studies including “Gaining control of schistosomiasis”, investigating the impact of different treatment strategies involving community-wide treatment (CWT), school-based treatment (SBT) and drug holidays (years in which a village did not receive praziquantel treatment) [16]. The gaining control study has been implemented in various African countries, including Tanzania, Kenya and Mozambique [16]. The SCORE study protocol and baseline characteristics have been described elsewhere [17, 18]. In the Mozambique study, the aim was to determine the strategy for the preventive chemotherapy that provides greatest reductions in prevalence and intensity of *S. haematobium* in school-aged children after four years of intervention in an area where baseline prevalence of infection was 25% or more. This article describes the baseline results pre-treatment and reports age, sex, prevalence and intensity of *S. haematobium* across all ages among 144 study sites selected for the study as well as potential environmental risk factors of local communities.

## Methods

### Study area and population

We selected 10 out of 17 districts of Mozambique’s northernmost province, Cabo Delgado, believed to be one of the least developed areas of the country. The province consists of lowlands adjacent to the Atlantic coast, with altitudes varying between 200 and 300 m above sea level. The climate is semi-humid with moderate rainfall of 800–1200 mm in the rainy season from November until April.

The area was chosen as the previous mapping had shown a high prevalence of *S. haematobium* and a low prevalence of *S. mansoni* (around 1%)*,* which met one of the SCORE country selection criteria of not working in areas with mixed infections [6, 17, 18]. The ten out of 17 districts included in this study were Metuge, Mecufi, Macomia, Ancuabe, Meluco, Quissanga, Namuno, Montepuez, Chiúre and Balama.

Eligibility for communities to be included in the study was determined by selecting only villages that had a primary school with a minimum of 100 children aged 9–12 years who attend school, since several arms of the study are school-based, and each community was randomised to any arm of the study. A cross-sectional survey was carried out in May and June 2011 to identify communities eligible to participate in the SCORE study. In total, 155 villages were selected at random across the ten districts. In each school, 50 children aged 13–14 years were randomly selected and tested for haematuria with dipsticks. The rationale for using 13–14-year olds in the eligibility survey was that children who test positive must be treated, therefore testing children aged 9–12 years and treating those infected could affect the subsequent study results, especially if prevalence is high [17]. Only those villages that demonstrated a microhaematuria prevalence of ≥ 21% by reagent strip testing (equivalent to ≥25% microscopic examination of filtered urine) were included in the study [17]. Since the rainy season lasts from November to April, baseline data was collected between July and October every year, commencing in 2011, to ensure there was no seasonal variation across the five years of data collection. Additional criteria for eligibility were that if two nearby communities share water sources and schools have an overlapping catchment area they were not considered separate communities for the study, and therefore only one of the schools were chosen. Secondly, communities were only considered that had not previously received preventive chemotherapy against schistosomiasis. All communities in this study were treatment naïve.

### Study arms

This study was a parallel cluster-randomised, intervention trial with six study arms [17, 18]. Communities received various combinations of CWT, SBT or drug holiday over a four-year period, with the final round of data collection carried out in Year 5. This paper only examines the baseline data pre-treatment.

### Sample size

To provide the sample size needed to complete all the planned arms, the baseline survey was conducted in 144 schools found eligible from July to October 2011. Initially, the school provided a list of the names, ages and sex of all children enrolled in each class. Primary schools have pupils at seven-year stages, Year 1 to Year 7. A year stage can, therefore, be defined as a group of pupils entering primary education at a common date. In each school 100 children aged 9–12 years (Years 2–7) and 100 children in the first class (Year 1) were then randomly selected from the list to participate. Where there were insufficient numbers of children in the age range, children were selected from outside of the age range. Also, parasitological data and information on the occupation of each sample were collected from 50 adults (aged 20–55 years) from each community.

### Urine examination

In each school, participants were given a wide-mouthed plastic bottle labelled with a specific identification number and asked to provide a single urine specimen. All samples were collected between 10 am and 2 pm and examined in the school by the field team straight away. Urine samples were vigorously shaken, 10 ml drawn into a syringe and pressed through a mesh nylon filter with a pore size of 20 μm (Sefar AG, Heiden, Switzerland). Two filtrations were carried out per urine sample. Both filters were placed on two separate microscope slides and, after adding a drop of Lugol, the filters were examined for *S. haematobium* eggs under a light microscope. All parasitological examinations were performed by experienced laboratory technicians. For quality control, around 10% of all microscope slides were re-examined by a senior technician. The intensity of infection was expressed as the number of eggs per 10 ml of urine filtered. For specimens of less than 10 ml, the volume of urine filtered was measured and the number of eggs per 10 ml calculated by extrapolation. If estimated counts were above 1000 eggs per 10 ml, they were truncated at 1000. The arithmetic mean of two filtrations taken from a single urine specimen was calculated to be the egg count of the child. A child was deemed egg positive if one or more eggs were found in any of the slides examined.

### Village and household level data

In addition to the parasitological survey, a village level questionnaire was asked to the community leaders to collect information that might affect schistosomiasis transmission. The questionnaire had sections on (i) general information (village name, population of village, number of households); (ii) main occupations of people in the village; (iii) local health facility information and whether they stock praziquantel; (iv) water contact sites (standing and flowing water); (v) water sources (drinking, washing and bathing); (vi) sanitation facilities in the village; and (vii) knowledge about schistosomiasis (heard about schistosomiasis, how to catch schistosomiasis, symptoms of schistosomiasis, how to prevent schistosomiasis, and name of the treatment).

### Data handling and analysis

Demographic data were collected on smartphones and uploaded to a dedicated database maintained on a central server (EpiCollect) hosted by Imperial College London. Laboratory data were collected on paper forms and entered into the smartphone retrospectively. Data cleaning and management were carried out by biostatisticians at SCI.

Data analysis was performed using R version 3.2 (URL https://www.r-project.org//) and STATA version 14 (StataCorp, College Station, TX, USA). The primary outcome of SCORE study is the change in prevalence and intensity of *S. haematobium* over the four years of intervention. Here, we present the findings of the baseline survey before treatment. Prevalence of *S. haematobium* infection from the eligibility survey was calculated from haematuria with dipsticks from a single urine sample from children aged 13–14 years.

From the baseline survey, infection categories for *S. haematobium* infection were defined per WHO thresholds: light infection (< 50 eggs/10 ml of urine) and heavy infection (≥ 50 eggs/10 ml of urine) [19]. Prevalence estimates were then calculated from the average egg count of two slides or egg count from one urine filtration (if only one 10 ml sample was taken) from three groups: (i) first-year students (aged 5–8 years); (ii) children aged 9–12 years; and (iii) adults (aged 20–55 years). Village-level prevalence was calculated for the prevalence of infection (0 for not infected, 1 for infected) and prevalence of heavy infection (0 for non-heavy infection, 1 for heavy infection). Prevalence of infection and heavy infection aggregated by other factors (e.g. age, sex) were calculated as arithmetic means of the infection categories. The Arithmetic mean of infection intensity was calculated for all subjects (including those with zero egg counts), which is a measure of community-level contamination potential. Although mean intensity was underestimated as egg counts were capped at 1000 eggs/10 ml, this was done consistently over the years, therefore, the trend in the change in intensity is believed to be valid. The association between sociodemographic characteristics (sexes, age groups, village and adult occupation) and infection was examined using Pearson *χ*^2^ tests, while one-way analysis of variance (ANOVA) was used to assess differences in intensities of infection. All tests and confidence intervals used the 5% level of significance.

## Results

### Baseline prevalence and intensity of *Schistosoma haematobium* infection

**Fig. 1:**
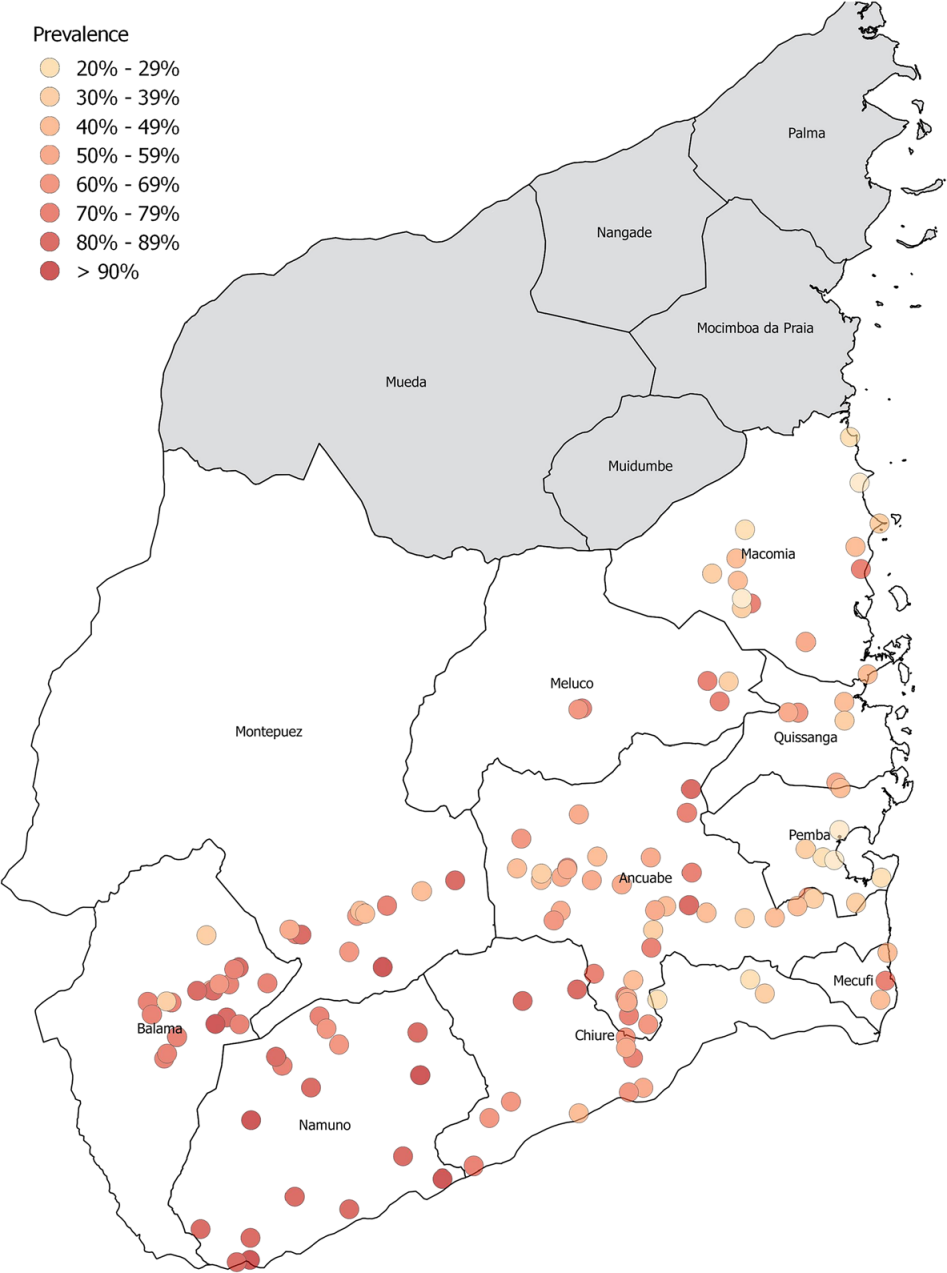
Prevalence of *S. haematobium* across all villages included in the SCORE study at baseline, in the ten districts of Cabo Delgado, Mozambique

Village level prevalence varied from 14.2% to 92.5%, with heavy prevalence ranging from 0 to 60%, across the 10 districts surveyed in Cabo Delgado (Fig. 1). Figure 1 is a map of the prevalence of *S. haematobium* across each village included in the study, in the 10 districts surveyed. Table 1 summarises the *S. haematobium* prevalence and intensity of infection pre-intervention by age group and sex. In total 151 out of 155 villages that were selected for the eligibility survey fulfilled the target endemicity cut-offs defined by SCORE with a microscopic haematuria prevalence of ≥ 21% and were therefore selected for the study. One village was dropped at random to achieve the target 150 villages. Unfortunately, the time taken to carry out the census, eligibility and cross-sectional survey at baseline took longer than expected and the data collection occurred in the rainy season for some villages. Six villages were inaccessible due to the rains and data was only collected in 144 villages.

**Table 1:** Prevalence and intensity of *S. haematobium* in first-year students (aged 5–8 years), 9–12-year-olds, 13–14-year-olds, and adults (aged 20–55 years) at baseline of a SCORE study in northern Mozambique

Age group (years)	No. of individuals examined	No. of individuals infected (%)^a^	Intensity of *S. haematobium* infection	
			Arithmetic mean intensity (eggs/10 ml)^b^	No. of heavily infected individuals (%)
Both sexes				
5–8	7463	4709 (63.1)	58.2	1440 (33.8)
9–12	7317	4873 (66.6)	71.9	1633 (33.9)
13–14^c^	5429	4010 (73.9)		
20–55	4259	1910 (44.8)	23.9	300 (15.8)
Total^d^	19,039	11,492 (60.4)	55.8	3373 (17.7)
Females				
5–8	3196	1922 (60.1)	44.7	517 (27.1)
9–12	3013	1890 (62.7)	54.4	547 (29.0)
13–14^c^	2276	1666 (73.2)		
20–55	1329	560 (42.1)	22.0	88 (15.9)
Total^d^	7538	4372 (58.0)	44.6	1152 (23.8)
Males				
5–8	4261	2784 (65.3)	68.1	923 (33.2)
9–12	4239	2946 (69.5)	84.9	1086 (37.1)
13–14^c^	3153	2344 (74.3)		
20–55	2924	1349 (46.1)	24.8	212 (15.8)
Total^d^	11,424	7079 (62.0)	63.12	2221 (26.7)

^a^For sexes combined, there was a significant difference in prevalence between age groups (*P* < 0.001) with the following sequence: 9–12 > 5–8 > 20–55 years (*χ*^2^ test). Within every age group, males had higher prevalence of infection than females in 5–8, 9–12 and 20–55 years (*P* < 0.001, *P* < 0.001, *P* < 0.01, respectively)

^b^For sexes combined, there was no difference between the two groups of children (age group 5–8 and 9–12 years), but intensity of infection among children was significantly higher (*P* < 0.001) than adults (one-way ANOVA). There was no difference between genders among school children, but male adults had higher intensities than females (*P* < 0.02)

^c^Eligibility results: prevalence of haematuria by dipstick from a single urine sample from children aged 13–14 years

^d^Total only includes the microscopic urine filtration data from the cross-sectional analysis, and excludes eligibility data that was collected in 13–14 year-olds using reagent strips only

The overall *S. haematobium* prevalence based on microhaematuria was 73.9% in 5429 children aged 13–14 years who participated in the eligibility survey (Table 1). A total of 19,039 individuals then provided urine samples at baseline, of which a total of 60.4% were infected. Examination revealed that of the 7463 children in the first year of school, 63.1% were infected with *S. haematobium* with significantly more boys (68.1%) than girls (60.1%) (*χ*^2^ = 23.2, *P* < 0.001). Among 7317 children aged 9–12 years tested, *S. haematobium* infection was found in 66.6% where the infection was higher in boys (69.5%) than girls (62.7%) (*χ*^2^ = 34.5, *P* < 0.001). A total of 4259 adults aged 20–55 years participated where 44.8% were found to be infected with significantly more males (46.1%) infected than females (42.1%) (*χ*^2^ = 6.35, *P* < 0.01). For sexes combined, the different age groups had significantly different prevalence (*χ*^2^ = 8.21, *P* < 0.01) with 9–12 years being the most infected, followed by first-year students and then adults.

In all age groups, the majority had light infections. A total of 17.7% had heavy infections of which 235 individuals (6.97%) had an egg count of 1000 eggs/10 ml or more. For the sexes combined, there was no difference between the two groups of children (first-year students and 9–12 years), but the intensity of infection among children was significantly higher (one-way ANOVA: *F*_(2,4154)_ = 8.013, *P* < 0.001) than adults. There was no statistically significant difference in intensity of infection between males and females among school children, but adult males had higher intensities than females (one-way ANOVA: *F*_(2,4152)_ = 6.197, *P* < 0.02).

**Fig. 2:**
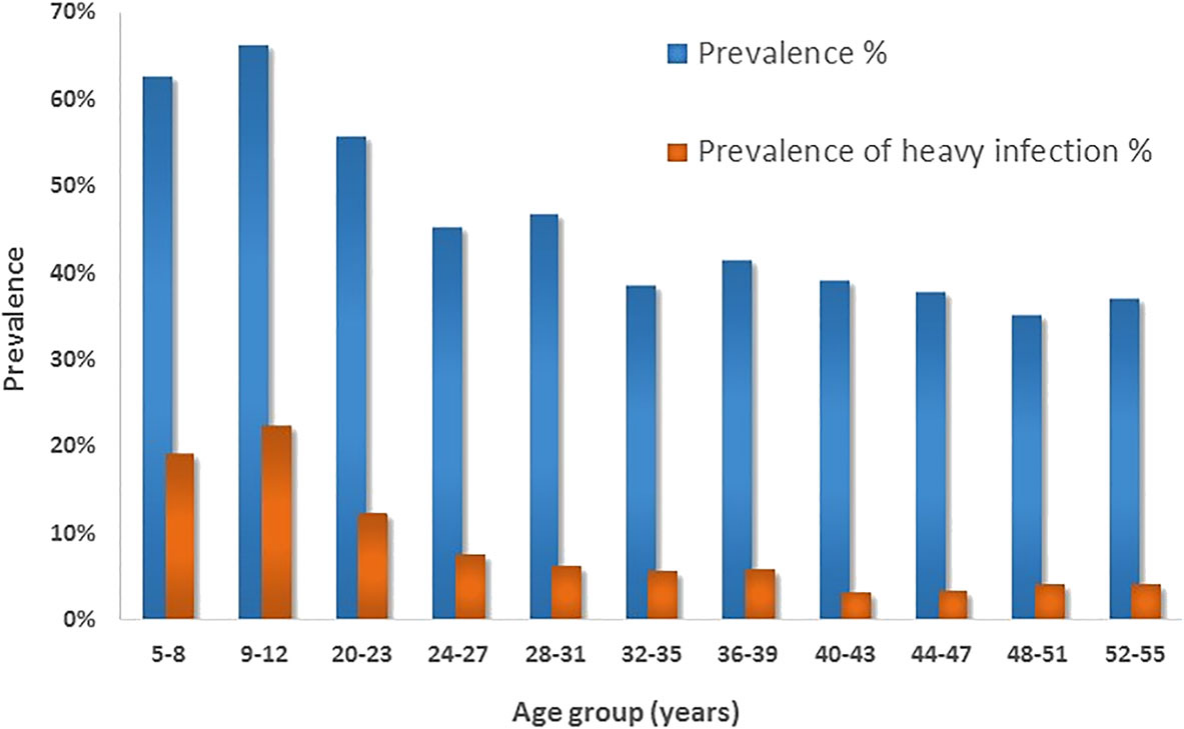
Prevalence of *S. haematobium* infection (dark blue) and heavy infection (red) by age group

The age profile for prevalence and heavy intensity infection with *S. haematobium* infection in Fig. 2 shows the breakdown by age, with a peak in infection rates among school-aged children and then a decrease into adulthood.

### *Schistosoma haematobium* infection and demographic and potential environmental risk factors of local communities

The prevalence and intensity of infection related to the main occupation among 4154 randomly selected adults is shown in Table 2. Most adults (95.6%) stated that farming was their main occupation. Overall there were no statistically significant differences between the prevalence of infection between professions. However, mean intensity of infection was higher among students although the numbers were not large enough to be significant (see Table 2).

**Table 2:** Prevalence and intensity of *S. haematobium* by occupation adults aged 20–55 years

Main occupation	No. of individuals examined	No. of individuals infected (%)	Intensity of *S. haematobium* infection	
			Arithmetic mean intensity of infection^a^	Heavy infection (%)
Farmer	3971	1805 (45.5)	46.7	276 (15.3)
Teacher	73	31 (43.7)	49.9	5 (16.1)
Student	45	21 (46.7)	103.4	12 (57.1)
Other	66	21 (31.8)	103.4	3 (14.3)
Total	4154	1877	49.8	297 (15.8)

^a^There was no statistical difference between prevalence or intensity of infection by occupation in the univariate logistic regression, controlling for sex and age

Table 3 summarises the potential environmental determinants of schistosomiasis infection in the study area. A village-level questionnaire was asked to 144 community leaders to collect information on potential risk factors associated with urogenital schistosomiasis. “Agriculture” was the main reported occupation (75.3%), although this was not expanded on in the questionnaire, and rice cultivation was practised in 23 villages (15.3%). Sixty-two villages had a health centre, however, only one of these stocked praziquantel. A total of 20.7% and 11.3% villages had a permanent and seasonal stagnant water body, respectively. Schistosomiasis risk behaviour such as washing (91.3%) and bathing (86.7%) in open water sources was high. Open urination was reported in most villages (83.3%), and use of pit latrines was reported in nearly all (98%) communities. Knowledge of schistosomiasis was relatively high (68.6%), as defined by the question “*Prior to today’s conversation, had you ever heard of [local term for schistosomiasis]?*”, However, correct knowledge of how the disease is acquired was low (23.2%) among those who had heard of the disease. Misconceptions such as the belief that schistosomiasis is transmitted through sexual contact were 12.3%. Most interviewees reported symptoms of schistosomiasis as pain on urination (76.5%), increased need to urinate (8.72%) and abdominal pain (3.36%). Knowledge about the existence of treatment against schistosomiasis was high (69.7%), although nearly all the respondents did not know the name of the drug (97.4%).

**Table 3:** Frequency of potential demographic, health-system related, and environmental risk factors associated with urogenital schistosomiasis reported by 144 communities in Cabo Delgado, northern Mozambique

Variable	Frequency	Percentage
Main occupation of inhabitants in the village		
Agriculture	113	75.3
Rice farming	23	15.3
Irrigation-based farming	7	4.7
Fishing	5	3.3
Local health facility		
Health facility open regularly	62	41.3
Does the health facility dispense PZQ	1	0.7
Water contact sites		
No. of villages with seasonal rivers	85	56.7
No. of villages with permanent rivers	76	51.0
No. of villages with permanent standing water body	31	20.7
No. of villages with seasonal standing water body	17	11.3
Water sources for drinking		
Well or borehole	133	88.7
Open surface water	125	83.3
Tap water	8	5.3
Water sources for washing/bathing		
Open surface water	137	91.3
Well or borehole	131	87.3
Tap water	9	6.0
Sanitation facilities		
Pit latrine	147	98.0
Bush/field	125	83.3
Improved latrine	10	6.0
Toilet	10	6.0
Heard about schistosomiasis	155	68.6
How do you catch schistosomiasis?^a^		
Do not know	79	50.1
Bathing in open water sources	36	23.2
Sexual	19	12.3
Drinking water	18	11.6
Symptoms of schistosomiasis^a^		
Pain on urination	114	76.5
Do not know	17	11.4
Increase need to urinate	13	8.7
Abdominal pain	5	3.4
How do you prevent schistosomiasis?		
Treatment	108	69.7
Do not know	31	20.0
Education	31	20.0
Other	12	7.7
What is the name of the treatment for schistosomiasis?		
Do not know	151	97.4
Praziquantel	4	2.6

^a^Knowledge among a total of 155 participants who had heard of urogenital schistosomiasis

## Discussion

Preventive chemotherapy with praziquantel is the current mainstay of schistosomiasis control, specifically the treatment of school-aged children as a function of prevalence level. To determine the best strategy to gain and sustain the control of *S. haematobium* in highly endemic areas, communities were selected to participate in a four-year cluster randomised intervention trial if there was a haematuria prevalence of ≥ 21% in 13–14-year-olds [16–18]. Here, we report the baseline parasitological situation in northern Mozambique before the onset of the study to assess the differential impact of school-based and community-based treatment.

Overall microhaematuria was high among the 144 villages examined, with 73.9% among 13–14 years by urine dipstick. When first-year students (aged 5–8 years) and 9–12-year-old children were examined with double filtration on a single urine sample in the baseline survey, the overall prevalence of *S. haematobium* was 63.1% and 66.6%, respectively. These findings were consistent with a national mapping survey carried out between 2005 and 2007 where schistosomiasis was found to be highly endemic among school children (aged 7–22 years) in Cabo Delgado province, with an overall prevalence of 57.9% of *S. haematobium*. The study highlights urine examination with reagent strips as a rapid diagnostic tool to identify high-risk areas for urogenital schistosomiasis consistent with previous studies [20–22].

As shown in other studies both gender and age had a significant effect on infection, with the highest prevalence and proportion of those heavily infected occurring in males and among 9–12-year age group with those same factors decreasing into adulthood [1, 23–28]. This finding may be explained by socio-cultural factors where an increased predisposition in boys to behaviours that expose them to the risk of infection such as swimming or bathing. This group will, therefore, be the focus for the monitoring and evaluating disease transmission from baseline through to Year 5 of this SCORE study.

This is the first study in Mozambique looking at infection rates among adults. Although children had higher levels of infection than adults, it was found here that adults had a high average infection of 44.5%. Per current WHO guidelines, children aged 5–14 years should be treated once every year in such high-risk communities (prevalence ≥50% measured by parasitological methods), as well as adults, considered “at-risk”. Occupation was recorded in two places in the baseline survey. When parasitological samples were taken from adults, they were asked their profession. Most adults (95.6%) stated that “farming” was their main occupation; however, there was no significant association between occupation and prevalence of infection. In addition, the village inventory questionnaire asked for village leaders about the main occupations of people in the village. Here, fishing and rice farming, which are widely shown to be a determinant of *S. haematobium*, were reported by 2% and 15.3%, respectively [29–32]. Since there is no single major cash crop in Cabo Delgado, agriculture has a more subsistence character with farmers selling small surpluses of crops such as cashew, groundnuts, cassava and maize.

The importance of contact with snail infested water and therefore with cercariae through bathing, combined with open urination, is acknowledged for governing the transmission of urogenital schistosomiasis [33, 34]. The community questionnaires were important in highlighting that knowledge of disease transmission is rudimentary, which has also been highlighted in knowledge, attitude and practices schistosomiasis survey that was conducted simultaneously to the SCORE study in the neighbouring province of Nampula in Mozambique [35]. Among our study population, use of open surface water was widely reported for washing and bathing, basic sanitation facilities such as pit latrines were reported to exist in most communities, yet open urination was widely practised. Furthermore, many villagers spend months at a time sleeping in tented accommodation when they were working in the ‘machambas’ (fields) with no access to sanitation. Although knowledge of the existence of schistosomiasis (translated into various local languages) was high, few understood the transmission cycle associated with spending time in open water sources. Misconceptions such as drinking dirty water and sexual intercourse were also described as ways to get schistosomiasis. Safe water, adequate hygiene and good sanitation interventions have shown to be successful in preventing schistosomiasis transmission elsewhere, and it would be recommended to complement the praziquantel treatment with supplementary measures such as health education and sanitation facilities [36, 37].

## Conclusions

In conclusion, the baseline results showed that Cabo Delgado Province in northern Mozambique is hyperendemic for urogenital schistosomiasis, particularly among school-aged children, and this risk appears to have remained unchanged over time [6, 7]. Prevalence rates were found to be greater than 50% in all districts, which highlights the need for annual praziquantel treatment in accordance with WHO guidelines. It also shows that prevalence differs by age and sex where the infection was also high among adults, which needs to be considered when planning programmatic schistosomiasis control efforts. In addition to preventive chemotherapy, access to safe water for washing and bathing, improved sanitation facilities and developing suitable health education tools could help tackle the burden of schistosomiasis. The aim of the SCORE project in Mozambique is to provide an evidence base for the national control programme to make informed decisions about preventive chemotherapy strategies to gain and sustain the control of *S. haematobium* infection. The results after a five-year period will demonstrate the cost-benefit impact of alternative approaches to schistosomiasis control through treatment of communities, schools, and treatment holidays.

## Abbreviations

CWT: Community-wide treatment
SAC: school-aged children
SBT: school-based treatment
SCORE: Schistosomiasis Consortium for Operational Research and Evaluation
STH: soil-transmitted helminths
WHO: World Health Organisation
